# Size-partitioning of an urban aerosol to identify particle determinants involved in the proinflammatory response induced in airway epithelial cells

**DOI:** 10.1186/1743-8977-6-10

**Published:** 2009-03-23

**Authors:** Kiran Ramgolam, Olivier Favez, Hélène Cachier, Annie Gaudichet, Francelyne Marano, Laurent Martinon, Armelle Baeza-Squiban

**Affiliations:** 1 Univ Paris Diderot, Paris 7, Laboratory of Molecular and Cellular Responses to Xénobiotics, Unit of Functional and Adaptive Biology affiliated to CNRS, 5 rue Thomas Mann, case courrier 7073, 75013 Paris, France; 2 Laboratoire des Sciences du Climat et de l'Environnement (LSCE/IPSL), Laboratoire CEA-CNRS-UVSQ, CEA Orme des Merisiers, 91191 Gif-sur-Yvette, France; 3 Laboratoire Interuniversitaire des Systèmes Atmosphériques (LISA), Université Paris 12, 61 avenue du Général de Gaulle, 94010 Créteil Cedex, France; 4 Laboratoire d'Etude des Particules Inhalées (LEPI), Ville de Paris, 11 rue George Eastman, 75013 Paris, France

## Abstract

**Background:**

The contribution of air particles in human cardio-respiratory diseases has been enlightened by several epidemiological studies. However the respective involvement of coarse, fine and ultrafine particles in health effects is still unclear. The aim of the present study is to determine which size fraction from a chemically characterized background aerosol has the most important short term biological effect and to decipher the determinants of such a behaviour.

**Results:**

Ambient aerosols were collected at an urban background site in Paris using four 13-stage low pressure cascade impactors running in parallel (winter and summer 2005) in order to separate four size-classes (PM_0.03–0.17 _(defined here as ultrafine particles), PM_0.17–1 _(fine), PM_1–2.5_(intermediate) and PM_2.5–10 _(coarse)). Accordingly, their chemical composition and their pro-inflammatory potential on human airway epithelial cells were investigated. Considering isomass exposures (same particle concentrations for each size fractions) the pro-inflammatory response characterized by Granulocyte Macrophage-Colony Stimulating Factor (GM-CSF) release was found to decrease with aerosol size with no seasonal dependency. When cells were exposed to isovolume of particle suspensions in order to respect the particle proportions observed in ambient air, the GM-CSF release was maximal with the fine fraction. In presence of a recombinant endotoxin neutralizing protein, the GM-CSF release induced by particles is reduced for all size-fractions, with exception of the ultra-fine fraction which response is not modified. The different aerosol size-fractions were found to display important chemical differences related to the various contributing primary and secondary sources and aerosol age. The GM-CSF release was correlated to the organic component of the aerosols and especially its water soluble fraction. Finally, Cytochrome P450 1A1 activity that reflects PAH bioavailability varied as a function of the season: it was maximal for the fine fraction in winter and for the ultrafine fraction in summer.

**Conclusion:**

In the frame of future regulations, a particular attention should thus be paid to the ultrafine/fine (here referred to as PM1) fraction due to their overwhelming anthropogenic origin and predominance in the urban aerosol and their pro-inflammatory potential.

## Background

Current levels of urban airborne particles are known to induce adverse health outcomes, including respiratory and cardiovascular diseases, and to be associated with an increased morbidity and mortality after short and long term exposure [1,2]. Amongst the biological effects of particulate matter (PM), the inflammatory responses of airway epithelial cells are of particular interest since they may represent one of the earliest short term effects of PM exposure, contributing to cardiopulmonary ill-health [3].

Since particle number, surface area and pulmonary deposition efficiency increase as particle size decreases, the fine and ultrafine aerosol fractions (commonly defined as PM_2.5 _and PM_0.1 _respectively) are expected to be responsible for the most significant health effects [4]. Moreover, ultrafine particles can easily penetrate the deep lung where macrophage effected alveolar clearance is less efficient than for larger particles [5]. Impaired clearance of particles from this site favours their interaction with epithelial cells and probably their transcytosis. However, there is still conflicting evidence from epidemiological studies and the limited number of toxicological investigations as to whether the fine aerosol fraction or the coarse one (PM_2.5–10_) is the most relevant fraction involved in human health effects [6-9]. The type of cells (macrophages versus epithelial cells, human versus rodent cells), the particle composition according to the sampling sites (content in metals, organic compounds, endotoxins...) as well as particle sampling mode and fractionation (filtration versus impaction, number of size fractions) could explain such discrepancies among toxicological studies.

The aim of this study is to compare the pro-inflammatory response of human airway epithelial cells exposed *in vitro *to different size fractions of Paris background aerosols. An experimental sampling and analytical methodology was developed based on the collection of the PM_0.03–0.17_, PM_0.17–1_, PM_1–2.5 _and PM_2.5–10 _aerosol fractions using 13-stage low pressure impactors (LPI). The aerosol mass, chemical composition and morphology were determined for each particle size class. The pro-inflammatory response was characterized by measuring the release by exposed cells of a cytokine, Granulocyte Macrophage-Colony Stimulating Factor (GM-CSF), cytokine release having been widely reported as the hallmark of PM toxicity [10,11]. GM-CSF is found to be a major regulator of both macrophages and neutrophils activation and survival in the lungs [12] and is involved in the maturation of dendritic cells [13]. Its increased release has been observed in the bronchoalveolar lavage of rodents exposed to diesel exhaust particles [14] as well as in the culture medium of bronchial epithelial cells exposed *in vitro *to diesel exhaust particles or PM [8,15,16].

Human bronchial epithelial cells (HBECs), one of the main target cells of particle with macrophages in the lung, were exposed to the different size-fractions of particles according to their proportion (here referred as isovolume exposure) or their actual abundance (isomass exposure) in the aerosol. Contribution of endotoxins in the cytokine release was investigated using a recombinant endotoxin neutralizing protein. In addition, cytochrome P450 1A1 (CYP1A1) activity was measured in human nasal epithelial cells HNECs) in order to gain insight into the bioavailability of organic compounds that have been previously shown to be involved in the GM-CSF release [17].

## Results and discussion

An experimental sampling based on the collection of the PM_0.03–0.17_, PM_0.17–1_, PM_1–2.5 _and PM_2.5–10 _aerosol fractions was developed using four using 13-stage Dekati low pressure impactors (LPI) running in parallel. In a previous pilot study with different LPI running in parallel we showed from mass determinations that particle recovery was reproducible [18]. We also set up a combination of two cell exposure strategies: cells were either exposed to the different size fractions according to their proportion in the aerosol (isovolume exposure) in order to try to imitate actual ambient exposure or as classically performed, at isomass exposure i.e. to the same particle mass This validation step was performed with a limited number of samples only, and focused on the biological effects of the two finest fractions of the aerosol. Promising results pointed to the necessary joined trans-disciplinary studies with parallel chemical and toxicological assays. Thus, seven short-duration samplings of size-segregated aerosols were performed either in winter and summer in order to investigate evolution of particle chemistry and biological reactivity according to atmospheric and seasonal conditions.

### Aerosol mass size distributions in Paris

**Figure 1:**
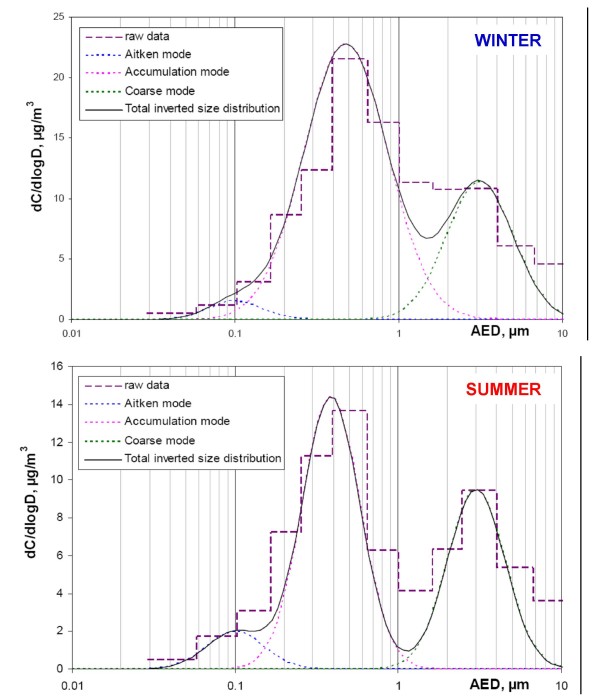
**Mass size distributions of the aerosol mass in winter and summer aerosols**. Using an inversion program described in Gomes et al., 1990 [38], raw data averaged for winter and summer separately (purple dashed lines) were inverted. The figure presents log-normal mass size distribution of the total aerosol (black plain line) and sorts out the three main contributions (Aitken, accumulation and coarse modes).

Mass concentration and contribution to PM_10 _are reported for each class-size fraction in Table 1. Results unambiguously underline the predominance of the fine fraction (PM_0.17–1_, 53 ± 13%). Data inversion for the seven LPI samples point to three main aerosol modes, namely the Aitken mode, the accumulation mode and the coarse mode with a geometric mean aerosol equivalent diameter (AED) of approximately 100 nm, 450 nm and 3 μm respectively. As presented in Figure 1, the threshold value between the accumulation and the coarse modes is found to be approximately 1.5 μm in winter and 1.2 μm in summer supporting our decision to study four different size fractions i.e. the ultrafine (PM_0.03–0.17_), fine (PM_0.17–1_), intermediate (PM_1–2.5_) and coarse (PM_2.5–10_) fractions. It has to be noted that aerosol collection with low-pressure cascade impactors may underestimate the smaller size-fractions of the aerosol mainly due to the evaporation of semi-volatile material from particles accumulated on the filters [19]. For PM2.5, this artefact was evaluated by comparison with TEOM/FDMS (artefact free) data and found to be of the order of 7%. However, particle loss underestimate is expected to be higher for ultrafine particles collected on the first stages of the impactor under significant vacuum conditions which suggests that this aerosol fraction could be still more reactive under isovolume condition.

**Table 1:** Description of LPI samplings in winter (W) and summer (S) 2005, mass concentrations and contribution to PM_10 _of the ultrafine, fine, intermediate and coarse fractions

			PM_0.03–0.17_		PM_0.17–1_		PM_1–2.5_		PM_2.5–10_	
	Start – stop (dd/mm)	Sampling duration and description	μg.m^-3^	%_PM10_	μg.m^-3^	%_PM10_	μg.m^-3^	%_PM10_	μg.m^-3^	%_PM10_
W1	11/01–12/01	22 h, 1 day	1.1	5	12.0	51	3.5	15	7.0	30
W2	12/01–14/01	47 h, 2 consecutive days	1.0	6	6.7	37	4.3	24	6.0	33
W3	14/01–17/01	65 h, 3 consecutive days	1.1	4	16.7	62	6.5	24	2.5	9
W4	24/01–03/02	98 h, stopped during 3 rainy days	1.2	7	11.2	63	3.0	17	2.4	14
										
S1	03/08–05/08	48 h, 2 consecutive days	0.7	5	5.5	36	2.8	18	6.4	41
S2	08/08–11/08	72 h, 3 consecutive days	1.6	12	6.9	50	1.9	14	3.4	25
S3	16/08–18/08	48 h, 2 consecutive days	1.3	9	10.3	70	1.3	9	1.8	12

### Morphology of individual particles

**Figure 2:**
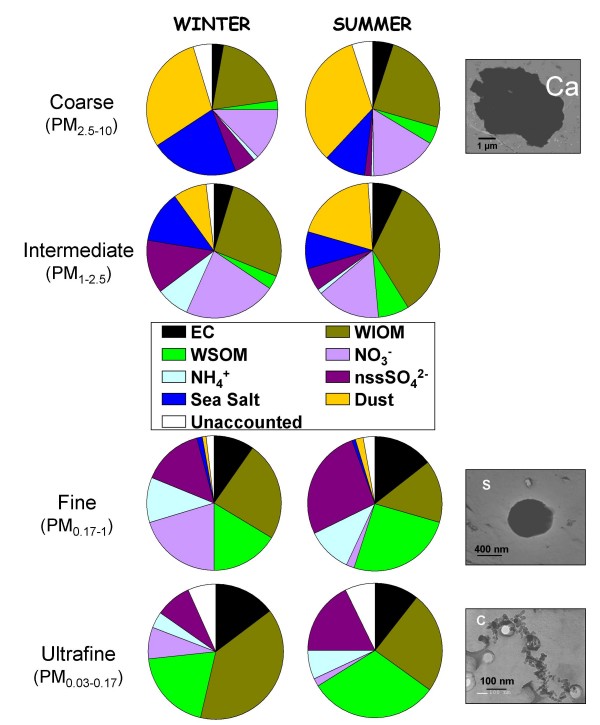
**Mean chemical composition in winter an summer and typical morphology for each size fraction**. Pies present the complete chemical composition of each size fraction (averaged for winter and summer experiments separately). EC: elemental carbon, WIOM: water-insoluble organic matter, WSOM: water-soluble organic matter, NO_3_^-^: nitrate, NH_4_^+^: ammonium, nssSO_4_^2-^: non-sea-salt sulphate, sea salt (calculated from sodium concentration), dust (mineral dust particles calculated from calcium concentration). Unaccounted is the difference between the aerosol fraction mass (gravimetric measurement) and sum of the major components EC, WIOM, WSOM, NO_3_^-^, NH_4_^+^, nssSO_4_^2-^, sea salt and dust. Morphology pictures (right side) present typical particles of size fractions and observed using TEM analysis.

Particle morphology was observed by Transmission electron microscopy (TEM). The different size-fractions differed greatly showing a tendency towards simplification for the smallest particles (Figure 2). The coarse aerosol displays a large variety of shapes including angular, prismatic and round particles, all of them mostly with a crystalline structure. Two types of particles were predominantly observed in the accumulation mode (fine fraction): round (sulphates) and micro-soot aggregates, whereas only fractal micro-soot aggregates could be observed in the ultrafine fraction. These chain-like aggregates are primarily of carbonaceous nature and clearly show an evolution of their shape factor (or length to width ratio) from 1.9 for the ultra-fine particles to 1.2 for the fine ones. These observations are in accordance with previous observations of the urban aerosol in Los Angeles by Xiong and Friedlander [20] or in Paris by Baulig et al., 2004 [10].

### Dose-dependence of the pro-inflammatory response

Pro-inflammatory cytokine release has been widely reported as a hallmark of PM toxicity [10,11]. Thorough mechanistic investigations have shown that such release results from oxidative insults triggering signalling pathways involved in increased expression of cytokine genes [21,22]. GM-CSF was chosen as a representative inflammatory cytokine as it is known to have pleiotropic effects in the inflammatory response and is considered as a potential therapeutic target to reduce the severity of inflammation in chronic obstructive pulmonary disease [12]. In addition in our pilot study, GM-CSF appeared as a sensitive biomarker of particle-induced proinflammatory response as substantial quantities were dose-dependently released by HBECs in response to fine or ultrafine particles at doses as low as 1 μg/cm^2 ^[18].

**Figure 3:**
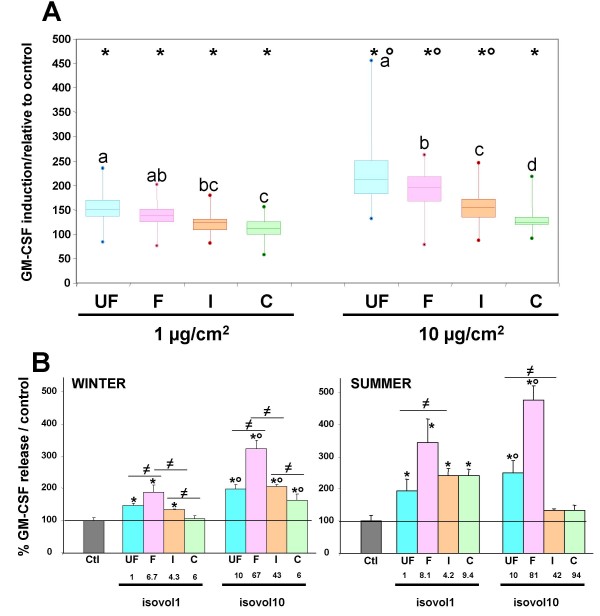
**Secretion of GM-CSF by 16HBE cells exposed for 24 hrs to the different size-fractions (UF: PM_0.03–0.17_, F: PM_0.17–1_, I: PM_1–2.5 _and C: PM_2.5–10_)**. a) **isomass exposure at 1 or 10 μg/cm^2^**: The data from seven size-segregated aerosol samplings were pooled and are presented as box plots. Each box plot is composed of 3 horizontal lines displaying the 25th, 50th and 75th percentiles. Minimal and maximal values are plotted as a dot. n = 82 for control, n = 20 for each size-fraction and dose. b) **isovolume exposure**: As the volumes of particle suspensions applied to cells were different for each experiment, average values could not be calculated. Representative experiments for winter (W) and summer (S) LPI samplings are presented. Volumes applied to cells were determined in order to get either 1 (isovol1) or 10 (isovol10) μg/cm^2 ^of ultrafine particles. For each fraction, the corresponding concentration in mass is given in μg/cm^2^. Similar results were obtained for each of the 7 samplings. Values are mean ± SD. n = 55 for control, n = 4 for each size-fraction. * different from control (p < 0.05), ° different from lower concentration (p < 0.001), different letters indicate significant difference between size-fractions within each concentration for isomass exposure (p < 0.001).

In the current study, two particle concentrations only were investigated for HBECs exposure. This is due to the limited amount of particles especially for the ultra-fine fraction. In all cases, without any cytotoxicity (data not shown), all size-fractions caused a significant GM-CSF release after a 24 hour exposure (Figure 3A) compared to unexposed cells. Whatever the season, the GM-CSF release was induced at 1 μg/cm^2^, a concentration that could be reached in airways of subjects with airway obstruction living in heavy polluted areas [23]. GM-CSF release increased as dose increased from 1 to 10 μg/cm^2 ^for the three finest fractions.

For isovolume exposures, as the volume of particle suspensions applied to cells were different for each sample, only two representative experiments are shown, one from a winter and another from a summer sample (Figure 3B). Again a dose-dependent GM-CSF release was observed for the two finest fractions.

### Size-dependence of the pro-inflammatory response

As shown in figure 3A, the pro-inflammatory response is more important for the finest fractions (PM_0.03–0.17 _and PM_0.17–1_) than for the larger ones.

For isomass exposures, independently of the season or the concentration (1 or 10 μg/cm^2^), GM-CSF release was maximal for the ultrafine fraction and decreased as the particle size increased (Figure 3A). These findings are in agreement with those obtained by Reibman et al. [8], reporting that only the smallest size fraction (AED < 0.18 μm) of New York PM induced a significant GM-CSF release by primary bronchial epithelial cells, and those of Huang et al. [24] showing an increased IL-8 release in response to Taiwan urban fine PM (AED <1 μm) exposure. This size-dependence of the pro-inflammatory response could be related to the higher particle number in the finest fraction. However, our results obtained from experiments conducted at isovolume exposures have shown that the highest GM-CSF release was always observed for the fine particle fraction rather than for ultrafine fraction (Figure 3B), although the fine fraction particle number is one order of magnitude less important that the ultra-fine fraction. Indeed, considering the average mass ratio (7:53) of the fine and ultra-fine fractions and assuming spherical particles with a density of 1.5 [25] and a diameter of 0.1 μm and 0.45 μm for the two fractions, a rough calculation indicates a ten-fold difference between the two fraction particle numbers. Thus other hypotheses should be considered to explain the highest GM-CSF release activity of the fine fraction during isovolume exposures. Among candidates, particle total surface area might deserve consideration. However, calculations indicate that for isovolume exposures, ultra-fine and fine fractions are likely to exhibit similar total surface areas.

During isovolume exposures, the more important effect of the fine fraction might be attributable to particle mass, fine fraction mass being about 8 times that of ultra-fine fraction. However, the ultrafine fraction was found to induce equivalent or higher levels of GM-CSF secretion than the intermediate or the coarse fractions (Figure 3B) although the ultra-fine fraction mass was found to be on average about 3 times or more lower than that of the intermediate or coarse fractions. Factors, other than particle number, surface area and or mass are likely therefore to be involved in PM induced GM-SCF secretion. These factors could be related to the chemical composition of each size fraction.

### Role of the chemical composition in the inflammatory response

Chemical compositions of the winter and summer size-segregated aerosols are displayed in Figure 2. The ultra-fine fraction displays a very peculiar chemical composition with the overwhelming presence of carbonaceous particles (EC, WIOM and WSOM) whereas the fine fraction although dominated by carbonaceous particles contains also a significant portion of secondary inorganic species (NO_3_^-^, NH_4_^+ ^and nssSO_4_^2- ^(non sea salt sulphate)), which mainly originate from anthropogenic activities. Conversely, the coarse fraction appears to be primarily impacted by natural particles (dust, sea salt and possibly carbonaceous bio-aerosols). The PM_1–2.5 _fraction, defined here as the intermediate fraction, is found to be a mixture of anthropogenic and natural aerosols. This chemical size-distribution may be characteristic of urban aerosols in Europe [26].

Influence of the chemical composition on GM-CSF activity was examined in light of linear regressions between concentrations of each major chemical species and GM-CSF release. Results are given in Table 2. Best correlation coefficients are obtained for the organic aerosol (r ≈ 0.6–0.7 for GM-CSF vs. OC in ultrafine, fine and intermediate fractions), and more specifically either its water soluble fraction WSOM (r ≈ 0.6 for GM-CSF vs. WSOM in fine and intermediate fractions) or its insoluble fraction WIOM (r ≈ 0.6 in ultrafine, fine and intermediate fractions). The major sources of (ultra-)fine carbonaceous aerosols in Paris are expected to be traffic all year long and anthropogenic or biogenic secondary organic aerosols in summer [27]. Significant residential biomass burning inputs may not be excluded in winter [28]. All these sources are likely to be responsible for the presence of harmful compounds in the finest aerosol fractions. However, due to the limited dataset available for this study, it is not possible to further estimate their relative contribution to the pro-inflammatory response.

**Table 2:** Correlation coefficients (r) obtained from the linear regressions between GM-CSF secretion and the mass of each chemical species within the different size fractions for isomass exposures (1 μg/cm^2 ^and 10 μg/cm^2^).

	**EC**	**OC**	**WIOM**	**WSOM**	**NO**_3_^-^	**NH**_4_^+^	**nssSO**_4_^2-^	**Sea Salt**	**Dust**
**UF**	**0,654**	**0,628**	**0,626**	0,470	0,587	0,078	0,242	0,308	0,312
**F**	0,573	**0,710**	**0,626**	**0,611**	0,352	0,247	0,236	**0,679**	0,530
**I**	**0,696**	**0,659**	**0,676**	**0,620**	0,485	0,546	**0,816**	0,168	**0,692**
**C**	0,152	0,105	0,192	0,184	0,301	-0,021	0,281	0,434	0,501

**Total**	**0,618**	0,505	0,412	**0,631**	0,069	0,282	0,350	-0,163	-0,163

n = 14 for each size fraction

**Figure 4:**
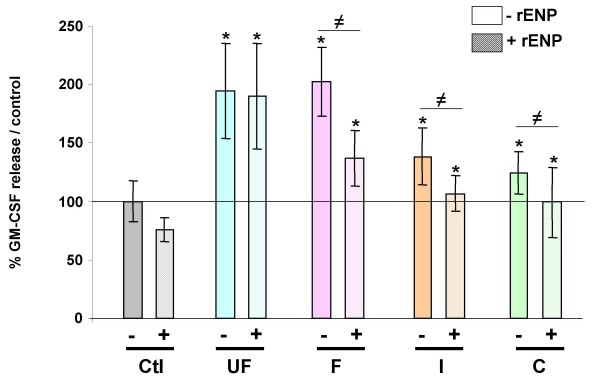
**Secretion of GM-CSF by 16HBE cells exposed for 24 hrs to 10 μg/cm^2 ^of the different size-fractions (UF: PM_0.03–0.17_, F: PM_0.17–1_, I: PM_1–2.5 _and C: PM_2.5–10_) in the presence (dashed bars) or absence (plain bars) of a recombinant endotoxin neutralizing protein (rENP)**. Data from four size-segregated aerosol samplings (W2, W4, S6, S7) were pooled. n = 55 for control, n = 8 for each size-fraction. * different from control (p < 0.05), ≠ significant difference between the presence or not of rENP.

Endotoxins are wall components of Gram (-) bacteria, that are frequently encountered within organic aerosols. They are known to have a high proinflammatory potential and have been previously shown to be preferentially associated with the coarse particulate fraction [29,30]. Their involvement in the GM-CSF release induced by the different size-fraction in HBECs was investigated by exposing cells to particles in presence or not of a recombinant endotoxin neutralizing protein (rENP) (Figure 4). rENP was shown to prevent GM-CSF release induced by the coarse PM fraction and to reduce GM-CSF release in response to fine and intermediate PM fractions (Figure 4). By contrast rENP did not reduce GM-CSF release in response to the ultrafine fraction, suggesting that the effects of ultrafine PM are independent of endotoxins.

**Figure 5:**
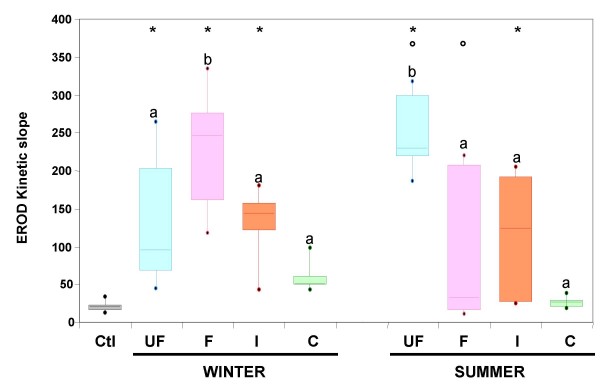
**CYP1A1 activity of human nasal epithelial cells exposed for 24 hrs to 10 μg/cm^2 ^of the different size-fractions (UF: PM_0.03–0.17_, F: PM_0.17–1_, I: PM_1–2.5 _and C: PM_2.5–10_) of winter and summer aerosol samplings**. The CYP 1A1 activity was measured for 40 min and the kinetic slope was determined. Kinetic slopes of four size-segregated aerosol samplings in winter and three size-segregated aerosol samplings in summer were pooled and presented as box plots. Each box plot is composed of 3 horizontal lines displaying the 25th, 50th and 75th percentiles. Minimal and maximal values are plotted as a dot. n = 8. * different from control (p < 0.05), ° different between seasons within a size-fraction (p < 0.001), different letters indicate significant difference between size-fractions within each season (p < 0.001).

Organic compounds are known to be involved in cytokine release induced by diesel exhaust particles (DEP) and urban PM_2.5 _[17,31] and a chemical separation of DEP organic compounds has shown that the polar fraction containing oxy-PAH is of primary importance in inducing the expression of oxidative stress sensitive genes [32]. In the present study the enzymatic activity of the cytochrome P450 1A1 (CYP1A1) was investigated as a biomarker of PAH bioavailability. PAHs are known to specifically induce the expression of CYP1A1 gene via their binding to the cytosolic aryl hydrocarbon receptor and subsequently to the xenobiotic responsive element flanking CYP1A1 gene. CYP1A1 gene expression is known to be specifically induced by diesel exhaust particles and their organic extracts [17]. As HBECs have no functional CYP1A1, although they express the CYP 1A1 gene and respond to PM exposure by increasing CYP1A1 expression [17,33], measurement of the CYP 1A1 activity was performed on primary cultures of human epithelial nasal cells. As shown in Figure 5, the highest CYP1A1 activity was obtained in response to the fine fraction in winter and to the ultrafine fraction in summer. This peak CYP1A1 activity observed in response to the fine fraction collected in winter may be related to the pattern of PAHs and/or to the elevated PAH concentrations within fine aerosols recorded during this season. Indeed, PAHs are relatively more abundant at wintertime due to thermal condensation of gaseous PAHs in the particulate phase [34]. For summer aerosols, the highest CYP1A1 activity observed in response to the ultrafine fraction could be explained by the fast formation of secondary organic aerosols via photochemical nucleation processes from the significant pool of traffic-generated PAHs [34].

### Implications for airborne particle regulations

This study thus indicates that the ultrafine and fine fractions of Paris background aerosols may induce significant pro-inflammatory responses in airway epithelial cells. Similar results have been previously obtained in other urban environments [8,24]. These findings reinforce the need for specific regulations concerning fine particles as already applied for a decade in the United States, but which are still under discussion in Europe.

Furthermore, the fine aerosol fraction is usually defined as particles with AED below 2.5 μm (PM_2.5_). However, chemical and biological results presented here suggest that PM_1 _might be more adequate than PM_2.5 _for the regulation of the fine aerosol fractions in Paris. As similar aerosol size distributions and chemical composition have been previously reported for other European cities [35], such observations might be helpful for optimizing the fine airborne particle policy in Europe. Representativity of results obtained with Paris aerosols obviously need to be assessed performing similar experiments with aerosols of different origins. In addition to ascertain the relevance of the pro-inflammatory response, other key pro-inflammatory cytokines should be investigated not only on bronchial epithelial cells but also on alveolar epithelial cells and macrophages.

Previous studies have reported contradictory results on the relative importance of the different aerosol size-fractions in PM pro-inflammatory responses. It might be suggested that for small and relatively clean cities, the more significant activity of the intermediate and the coarse fractions [9,36,37] is apparently due to endotoxins and metals whereas in bigger cities such as New York or Paris [[8,24], this study] the predominant effect of the finest fraction in the pro-inflammatory response of bronchial cells is related to the presence of organic compounds. In large cities of developed countries, this effect could be due to the prominent importance of traffic particles, and reinforces the idea that the anthropogenic organic fraction of the aerosol represents one of the main factors for adverse health effects of urban aerosols. In the frame of future regulations, a particular attention should thus be paid to these fine and ultrafine aerosol fraction.

## Conclusion

Combining in vitro toxicological studies and a thorough chemical characterization of an urban background aerosol according to its size fractions, we provide evidence that the finest fractions are the most prone to induce a pro-inflammatory response in airway epithelial cells in relation with their chemical composition. Furthermore, our work strongly suggests that in Paris and similar urban wards in Europe, PM1 is more representative of the fine aerosol than PM2.5 in the context of airborne particle policy dedicated to human health effects.

## Methods

### PM sampling

Ambient aerosols were collected on the terraced roof (17 m above ground) of the Laboratoire d'Hygiène de la Ville de Paris (Paris, 13^th ^district), a site corresponding to an urban background station of the AIRPARIF air quality monitoring network. The experimental set up comprised four Dekati^® ^13-stage low pressure cascade impactors (LPI) running in parallel. Consistency of aerosol mass partition results obtained from the parallel impactors was checked to be satisfactory [18]. Two LPIs, equipped with 25 mm-diameter polycarbonate membranes (Nuclepore AOX), were dedicated to biological experiments, while one other devoted to mass measurements and to chemical analyses (including major ions and water-soluble carbon) was mounted with Teflon filters (Zefluor, Pall^®^) and the fourth LPI designated for elemental and organic carbon analyses (EC and OC) was mounted with quartz fiber filters (QMA, Whatman^®^). Four samplings were performed during the winter season (W1, W2, W3 and W4) and three during the summer season (S1, S2 and S3) 2005 (Table 1). Additional samplings of short duration (~20 min) were performed in winter and summer for transmission electron microscope (TEM) observations. For this purpose, an impactor was mounted with alphanumeric copper electron microscope grids of 200 mesh covered with pre-metallized polycarbonate membranes (porosity 0.2 μm, Whatman^®^).

### Size definition of the four size classes of interest

**Figure 6:**
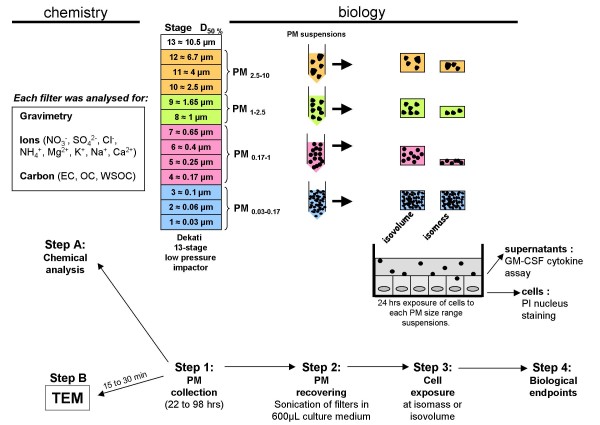
**Sampling and experimental strategy overview**. Particles were sampled with four 13 stage low pressure Dekati impactors running in parallel for 22 to 98 h according to samples (Step 1). For chemical analysis (Step A), gravimetry, ions and carbon content were determined on each stage filter. For biological analysis, filters were gathered to constitute 4 PM-size fractions (PM_0.03–0.17_, PM_0.17–1_, PM_1–2.5_, PM_2.5–10_). They were briefly sonicated directly in 600 μL culture medium (Step 2). HBECs were exposed for 24 hrs to the different PM-size fractions (Step 3) either at the same volume of particle suspension (isovolume exposure) or at the same concentration of particle suspension (isomass exposure). After exposure, GM-CSF release was measured in the culture medium and cell viability was assessed using a propidium iodide (PI) assay (Step 4). Specific short samplings (20 min) were performed to collect particles on specific supports for transmission electron microscopy (TEM) observations (step B).

Fine particles are commonly defined as particles with aerodynamic equivalent diameters (AED) below 2.5 μm. For Paris aerosols, a pilot study indicated that the AED threshold between fine and coarse particles might actually be around 1 μm [18]. For this reason, four size classes were investigated: PM_0.03–0.17 _(ultrafine (UF), LPI stages 1–3), PM_0.17–1 _(fine (F), stages 4–7), PM_1–2.5 _(intermediate (I), stages 8–9), and PM_2.5–10 _(coarse (C), stages 10–12) as presented in Figure 6 (step 1).

### Determination of the aerosol mass and chemical composition of each particle size class. Figure 6 (step A)

The aerosol chemical closure was achieved for each size class, following the new procedure detailed by Guinot et al [37]. Briefly, gravimetric measurements were performed on the teflon or polycarbonate filters using a microbalance (Sartorius model MC21S). The mass log-normal distribution was obtained for each LPI sample using a data inversion program [38]. Chemical analyses comprised the determination of major ions and carbon components. Extracts of teflon filters were obtained by exposure to ultra-sounds in 15 mL of ultra-pure water during 45 minutes. These extracts were analysed to determine their anion (NO_3_^-^, SO_4_^2-^, Cl^-^) and the cation (NH_4_^+^, Mg^2+^, K^+^, Na^+^, Ca^2+^) composition by ion chromatography (Dionex DX-600 model). Sea salt and dust mass concentrations were calculated using Na^+ ^and Ca^2+ ^concentrations respectively. The water soluble organic carbon (WSOC) content was obtained from the same extracts using a TOC analyser (Sievers 900 model). Two elemental and organic carbon fractions (EC and OC) were obtained from the quartz fiber filters following the 2-step thermal method described in Cachier et al. [39]. From these measurements water insoluble organic carbon fraction, WIOC is calculated as the difference between OC and WSOC. Water soluble organic matter (WSOM) and water insoluble organic matter (WIOM) could be obtained using adequate conversion factors for the fine and coarse particles. Following Turpin and Lim [40]: the following values were adopted:

WSOM/WSOC = 2.1 for all sizes whereas WIOM/WIOC = 1.3 for fine particles and WIOM/WIOC = 1.8 for coarse particles.

### Individual particle characterization. Figure 6 (step B)

Transmission electron microscope (TEM) observations and analyses were conducted using a TEMSCAN (JEOL^® ^100 CX II) with a spatial resolution of 0.2 nm. The microscope was fitted with an energy dispersive X-ray analyser for chemical microanalysis (PGT Prism 2000) which permits the detection of elements with an atomic number superior or equal to six (carbon) and with a digital camera (GATAN ERLANGSHEN-780). The pictures were taken from X36000 for the ultrafine particles to X3600 for the coarser ones. A semi-automated image analysis was performed (Microvision-Histolab) to document the particles morphology, size and shape factor.

### Reconstitution of particle suspensions for toxicological experiments

As illustrated in Figure 6 (step 2), particle suspensions of the different size-fractions were obtained by sonication (3 × 10 sec, 60 Watts) of sampled filters directly into 600 μL of cell culture medium (DMEM/F12, Invitrogen^®^). The efficiency and reproducibility of this extraction protocol have been demonstrated by Ramgolam et al. [18], especially we checked the efficient detachment of particles from the filter by scanning electron microscopy. Particle suspensions were stored at -20°C until use and were again sonicated (3 × 10 sec) just before dilution in the culture medium for cell exposure.

### Cell cultures

Either human bronchial or nasal epithelial cells (HBECs and HNECs) were exposed to sampled size-segregated particles according to the biological endpoints investigated.

HBECs used in this study correspond to the subclone 16HBE14o- line kindly provided by Dr. D. C. Gruenert (San Francisco, California, USA). Cells were grown in DMEM/F12 culture medium supplemented with penicillin (100 U/mL), streptomycin (100 μg/mL), L-glutamine (1%), fungizone (0.125 μg/mL) and UltroserG (UG, 2%). 20,000 cells/cm^2 ^were seeded on collagen (type I, 4 μg/cm^2^) coated 24-well plates (Costar^®^) and cultured in humidified 95% air with 5% CO_2 _at 37°C. After two days, the subconfluent cultures were deprived of UG for 4 hrs and exposed to the particles of the different PM-size fractions for 24 hrs.

Primary cultures of human nasal epithelial cells (HNECs) taken from nasal turbinates obtained from patients undergoing turbinectomy were established according to the method previously described by Million et al. [41]. For particle exposure, HNECs were seeded in 48-well plates at 20.000 cells/well and cultured for 2–3 days in DMEM/F12 containing growth factors (insulin (5 μg/ml), hydrocortisone (0.5 μg/ml), epinephrine (0.5 μg/ml), triiodothyronine (6.5 ng/ml), transferrin (10 μg/ml), human epidermal growth factor (0.5 ng/ml), gentamicin:amphotericin B (50 μg/ml:50 ng/ml) and bovine pituitary extract (0.13 mg/ml).

### Cell culture exposure to size-fractionated particles

Exposures were conducted following two different strategies (Figure 6, step 3). (i) For each size fraction, cells were exposed to suspensions containing the same concentration of particles, referred here as an isomass exposure that is the most classical exposure when comparing particle samples. In this study, isomass exposures were conducted at 1 and 10 μg/cm^2^, corresponding to 5 and 50 μg/mL. (ii) For each size fraction, cells were exposed to the same volume of particles in suspension. This exposure strategy, referred here as isovolume exposure, led to cells being exposed to the different size fractions according to their relative proportion in ambient air during sampling. The volumes applied to cells were calculated in order to get either 1 or 10 μg/cm^2 ^of ultrafine particles and consequently were different for the seven samplings.

### Cell viability assay

After 24 hrs of exposure (Figure 6, step 4), cell viability was immediately evaluated by Propidium Iodide (PI) nuclear staining to reveal cell membrane damage. Cells were dissociated using trypsin-EDTA then 5 μg/mL of PI (Sigma^®^) was added and the percentage of cells that incorporated PI was assessed in a sample of 10,000 cells using flow cytometry (CyAn LX, DakoCytomation^®^) with an excitation wavelength of 488 nm and an emission wavelength of 635 nm.

### GM-CSF assay

After 24 hrs of exposure (Figure 6, step 4), the culture medium was removed, centrifuged to eliminate particles and stored at -80°C until GM-CSF measurement. The GM-CSF content of cell supernatants was measured using a human GM-CSF Duoset ELISA development system kit according to the manufacturers' instructions (R&D systems Europe). Color development was measured at 450 nm with a microplate photometer MR5000 (Dynex technologies).

### Endotoxin analysis

The impact of endotoxins on GM-CSF release was evaluated using a recombinant endotoxin neutralizing protein (rENP), consisting of an 12.2 kD protein purified from the amebocytes of the horseshoe crab, *Limulus polyphemus *(Cape Cod Associates^®^). This protein neutralizes the bioactivity of lipopolysaccharide (LPS) when used in a 1:1 ratio (weight) of ENP/LPS. rENP was diluted in water and used at 2 μg/mL [29].

### Evaluation of CYP1A1 activity with the Ethoxy-Resorufine O Deethylase assay

The biological effect of anthropogenic organic aerosols was assessed by measuring cytochrome P450 1A1 (CYP1A1) enzyme activity using the Ethoxy-Resorufine O Deethylase (EROD) assay. The HBECs used in this study lacked functional CYP1A1 activity, therefore this measurement was undertaken using HNECs. Cells were then deprived of growth factors for 4 hrs and exposed to particles (10 μg/cm^2^) in growth factor free DMEM/F12 medium for 24 hrs. Cells were washed with phosphate buffer saline (Invitrogen^®^) and incubated with DMEM/F12 containing 5 μM ethoxyresorufine and 2 mM salicylamide. Ethoxyresorufine is metabolized by CYP1A1 leading to the formation of resorufin, which is fluorescent. Kinetic fluorescence measurements were made with a microspectrofluorimeter (Fluostar galaxy, GMB^®^) with an excitation wavelength of 530 nm and an emission wavelength of 590 nm for 40 min. The EROD kinetic slope was determined over 40 min.

### Statistics

For each biological experiment, particle exposures were carried out in 3/4 replicates. Statistical analyses were achieved using one-way ANOVA followed by the Student-Newman-Keuls test.

## Competing interests

The authors declare that they have no competing interests.

## Authors' contributions

KR carried out toxicological studies, data analysis and performed the statistical analysis. OF contributed to particle sampling campaigns, carried out chemical analysis, participated in data evaluation as well as writing the paper. HC conceived the study, supervised chemical analysis and helped to draft the manuscript. AG contributed to the research idea, the design and performance in the study. FM contributed to the research idea. LM conceived the design of the study, participated in particle sampling campaigns, performed particle morphological characterization, and participated in coordination and data analysis. ABS conceived the design of the study, participated in the coordination and data analysis and wrote the manuscript. All authors read and approved the final manuscript.
